# Association between workplace violence from patients and the mental health status of healthcare workers in Zhuhai China: a cross-sectional study

**DOI:** 10.3389/fpubh.2024.1441389

**Published:** 2025-01-07

**Authors:** Ying Zheng, Xuping Li, Yajun Sun, Chun Mao, Jiaju Huang, Jingya Li, Guangchuan Zhang, Ning Wei, Xiaohui Wang, Yongyong Teng

**Affiliations:** ^1^Xiangya School of Public Health, Central South University, Changsha, Hunan, China; ^2^The Third People's Hospital of Zhuhai, Zhuhai, Guangdong, China; ^3^Zhuhai Mental Health Association, Zhuhai, Guangdong, China

**Keywords:** workplace violence, patient, depression, insomnia, healthcare worker

## Abstract

**Objective:**

Workplace violence (WPV) poses a serious occupational risk. This study aims to explore the association between WPV from patients and the occurrence of insomnia, depression, and anxiety among healthcare workers.

**Methods:**

Information about the WPV from patients was collected by a self-designed questionnaire. Generalized Anxiety Disorder-7 (GAD-7), Patient Health Questionnaire-9 (PHQ-9), and Insomnia Severity Index (ISI) were used for the assessment of mental health. Logistic regression was used to explore the association between WPV from patients and insomnia, depression, and anxiety. Mediation analysis was used to evaluate the mediation effect of depression and anxiety on the relationships between WPV from patients and insomnia.

**Results:**

Of 10,413 included healthcare workers, 40.05% experienced verbal violence, 6.44% experienced physical violence from patients in the past year. There is a significant association between verbal violence and insomnia (OR = 1.780, 95% CI: 1.591–1.990), depression (OR = 1.823, 95% CI: 1.640–2.026), and anxiety (OR = 1.831, 95% CI: 1.606–2.087), as well as physical violence (insomnia: OR = 1.220, 95% CI: 1.002–1.481; depression: OR = 1.274, 95% CI: 1.052–1.540; anxiety: OR = 1.316, 95% CI: 1.058–1.630). Moreover, depression and anxiety mediated the relationship between WPV and insomnia, the mediated proportion was 62.21% in the association between verbal violence and insomnia, and 60.22% in the association between physical violence and insomnia.

**Conclusions:**

The association between WPV from patients and heightened risks of mental health issues emphasizes the necessity of supportive work environments. Recognizing the mediating role of depression and anxiety stresses the significance of tailored mental health training for healthcare staff.

## Introduction

Workplace violence (WPV) poses a significant occupational hazard for healthcare workers and has become a focal point of concern (1, 2). According to the International Labor Organization (ILO), about a quarter of WPV incidents occur in medical institutions (3). In the United States, 70%−74% proportion of WPV occurs in healthcare institutions (4). From 2012 to 2015, the overall rate of WPV affecting hospitals increased by 23% (5). Patients are the most common perpetrators of WPV among these institutions (6). A survey conducted in 2014 on WPV in hospitals in the United States found that WPV from patients accounted for 75% of cases (7). The prevalence of WPV in medical home visits after work in Australia was 47.1% in 2017, and just over half (51.8%) of the cases came from patients, verbal violence was the commonest (48.3%) (8). The same is true in China. According to data from the China Judgment Online System, the proportion of healthcare workers experiencing WPV by patients increased from 35% in 2013 to 73% in 2016 (9). The alarming escalation of WPV underscores its detrimental impact on the wellbeing of healthcare professionals, leading to both physical and psychological harm.

Studies have established a link between WPV and adverse outcomes such as depression, anxiety, and insomnia among workers (10–14). The WPV may be an important factor causing depression and anxiety among workers (15). Healthcare workers, given the demanding nature of their profession, are particularly vulnerable to these mental health conditions, with depression prevalence ranging from 21.53% to 32.77% in high-income countries in 2015 (16). WPV among healthcare workers has been closely linked to depression and anxiety. Zafar et al. found that healthcare workers who have been subjected to WPV exhibit higher rates of burnout and are more susceptible to depression and other psychological disturbances (17).

Persistent depression and anxiety can disrupt sleep patterns, leading to insomnia, which further exacerbates daytime functioning issues and contributes to a host of negative health outcomes, including fatigue, irritability, and reduced work performance (18, 19). Various work-related stressors are known to play a role in the development of insomnia, with WPV being a particularly prominent stressor. Individuals who have experienced WPV are 2.35 times more likely to experience poor sleep quality than those who have not encountered such violence (20). Moreover, Wang et al. study underscores the detrimental effects of WPV, emphasizing its role in job burnout and diminished work efficiency, with insomnia serving as a key mediating factor in this relationship (21).

Addressing the complex interplay between WPV, mental health disorders, and sleep disturbances is crucial in safeguarding the wellbeing of healthcare professionals and optimizing their performance in an inherently challenging work environment (22, 23). However, current research has some limitations. Some studies tend to focus exclusively on the prevalence of WPV in specific clinical departments, such as emergency and psychiatric units, neglecting other departments like radiology where staff also interact closely with patients. Furthermore, there is a scarcity of analysis differentiating the impacts of verbal and physical violence within the context of WPV. Many studies independently analyze the effects of WPV, and the precise connections between WPV and insomnia, depression, and anxiety remain inadequately understood. Addressing these limitations could contribute to a more comprehensive understanding of the complex interplay between WPV and mental health outcomes among healthcare workers.

This study aims to explore the associations between WPV from patients and the mental health of healthcare workers, with a specific focus on the pathways through which verbal and physical violence impact depression, anxiety, and insomnia. By delving into these relationships, we aim to provide a scientific basis for developing more effective prevention and intervention strategies to protect the mental and physical wellbeing of healthcare workers and enhance work efficiency.

## Materials and methods

### Study design and participants

This study conducted a cross-sectional survey of clinicians, nurses, and paramedical staff in secondary and tertiary medical institutions and community health service centers in Zhuhai, China in October 2023, including radiology, anesthesiology, and other departments. This study was conducted in accordance with the Declaration of Helsinki, and was approved by the ethics committee of Zhuhai Third People's Hospital.

### Data collection

Data were collected using an online questionnaire consisting of 64 questions that included demographic information, job-related information, and three standardized scales for depression, anxiety, and insomnia. The link was sent to participants via WeChat social media group chats organized by the institution. The foreword of the questionnaire provides an introduction to the research, and participants can choose to click “agree” to continue the survey or click “disagree” to exit the survey, so as to ensure the informed consent of participants. To ensure the quality of the survey, we set some quality control questions (including the answer to the capital of China and the average working hours/days per day/week). Incorrect or unreasonable answers were excluded from the analysis. Fifty-one medical institutions in Zhuhai met the survey requirements, with a total of 17,279 healthcare workers. Thirteen thousand eighty-six questionnaires were completed, with a coverage rate of 75.73%. Finally, 10,413 were included in the study, with a pass rate of 79.57%, and the average time for participants to complete the questionnaire was 703 s.

### Assessment of WPV

The frequency of physical and verbal violence from patients was evaluated using a customized questionnaire. Participants were queried with the question, “In the past year, how many times have you faced physical violence from patients such as hitting, kicking, pushing, biting, or hair-pulling?” Response choices included “None,” “1–3 times,” “4–6 times,” “7–9 times,” and “10 times or more.” Similar questioning was applied to verbal violence, with participants asked, “In the past year, how many times have you faced verbal violence from patients in the form of threatening language, humiliation, or other disrespectful remarks while on duty?” Participants selected one of the aforementioned five response options.

### Assessment of insomnia

The validated Chinese version of the Insomnia Severity Index (ISI) was employed for the assessment of insomnia (24, 25). This 7-item self-report questionnaire measures the severity and impact of insomnia across multiple domains, including sleep onset, sleep maintenance, early morning awakening problems, sleep dissatisfaction, interference with daytime functioning, perception by others, and distress caused. Each item was assessed on a 5-point Likert scale ranging from 0 to 4, where 0 indicates the absence of the issue and 4 indicates a severe problem. The total score was from 0 to 28, and a score of 15 or greater was considered insomnia.

### Assessment of depression

The validated Chinese versions of the 9-item Patient's Health Questionnaire (PHQ-9) were utilized to assess depressive symptoms over the previous 2 weeks (26, 27). Participants rated each item on a 4-point Likert scale, with response options of “not at all,” “several days,” “more than half the days,” and “nearly every day” corresponding to scores of 0, 1, 2, and 3, respectively. The highest potential score on the PHQ-9 was 27, participants with a total score of ≥10 are considered to have depression.

### Assessment of anxiety

The validated Chinese versions of the Generalized Anxiety Disorder Questionnaire (GAD-7) were employed to evaluate anxiety symptoms experienced during the preceding 2 weeks (28, 29). Analogous to the PHQ-9, the score for each item on the GAD-7 ranged from 0 (not at all) to 3 (nearly every day). The maximum achievable score on the GAD-7 was 21, participants with a total score of ≥10 are considered to have anxiety.

### Covariates

The selection of covariates is based on previous literature (30, 31). Sociodemographic factors included age (18–29 years, 30–44 years, and 45–60 years), gender (male and female), marital status (married, unmarried/divorced/widowed), education attainment (high school or below, university, and postgraduate or above). Work-related factors included monthly income (< 5,000 yuan, 5,000–10,000 yuan, 10,001–20,000 yuan, >20,000 yuan), income satisfaction (relatively satisfied, average, not satisfied), workload (not heavy, average, heavy), and job category (clinicians, nurses, paramedical staff). Additionally, data were collected on the participants' interpersonal relationships, the number of mental health lectures attended in the past year, and the experience of major negative life events such as divorce, unemployment, bereavement, and healthy lifestyle as covariates. Interpersonal relationships were synthesized by investigating participants' relationships with leaders, colleagues, and family members. Participants rated each relationship from 1 to 5, with the better the relationship, the better the score. A total score of < 9 was considered poor, < 12 was considered average, and ≥12 was considered good. The study considered four lifestyle behaviors based on prior research: smoking, drinking, physical activity, and BMI (32–34). Non-current smoking and non current drinking were defined as low-risk behavior. BMI was calculated from participants' self-reported height and weight and low-risk BMI was defined as >18.5 and < 23.9. Physical activity was evaluated by asking participants about moderate-intensity exercise frequency over the last 3 months. Exercising at least 30 min, five or more times per week was considered low-risk behavior. Participants received 1 point for each low-risk behavior, contributing to an overall healthy lifestyle score ranging from 0 to 4, where a higher score denoted a healthier lifestyle.

### Statistical analysis

The basic characteristics of the participants were described using the percentage for categorical variables and means ± standard deviations for continuous variables. Logistic regression models were constructed to assess the association between different types and frequencies of WPV from patients and insomnia, depression, and anxiety. Three models were developed for analysis. Model One adjusted for age, gender, education attainment, and marital status. Model Two further included work-related factors including income satisfaction, monthly income, workload, and job category. Model Three additionally added interpersonal relationships, negative significant life events, healthy lifestyle scores, and mental health lectures. In these models, physical violence and verbal violence were adjusted to each other. Further, we conducted an individual model to evaluate the effect of the interaction between verbal violence and physical violence on the basis of Model Three.

The mediation effect of depression and anxiety on the relationships between WPV from patients and insomnia of healthcare workers was evaluated. Indirect, direct, and total effects for each mediator were computed by combining the mediator and outcome models with the adjustment of all the covariates in Model Three. Further subgroup analyses were performed to examine potential differences in the relationship between verbal and physical violence and their interaction and depression and anxiety based on gender and job category. Additionally, we evaluated the odd ratio (OR) for participants exposed to various violence types versus those not exposed to any violence. Data cleaning and regression modeling were performed using the R statistical software (version 4.3.2), and mediation analysis was performed using IBM SPSS version 26.0.

## Results

There were 13,086 healthcare workers who completed the assessment. After excluding participants under 18 or over 60 years old (*n* = 93), those with incorrect responses on quality control questions (*n* = 2,246), and those lacking complete socio-demographic information (*n* = 334). A total of 10,413 healthcare workers were ultimately included in this study.

### Characteristics of included participants

Among 10,413 participants, 1,900 (18.25%), 2,364 (22.70%), and 1,335 (12.82%) were with insomnia, depression, and anxiety, separately. The majority of the participants were female (74.12%), aged 30–44 years (50.85%), married (63.32%), and possessed a postgraduate or higher education level (81.28%). Most of the participants were nurses (48.89%) with a monthly income ranging from 5,000 to 1,0000 yuan (45.42%). About 34.62% of the participants perceived their workload as heavy, and 27.00% were not very satisfied with their income. Only 25.22% of the participants had received mental health lectures, and 11.04% had experienced negative life events in the past year. There were 4,170 participants (40.05%) experienced verbal violence, 671 participants (6.44%) experienced only physical violence. The detailed characteristics of participants are shown in Table 1.

**Table 1:** The characteristics of the participants included in the study.

	**Total**	**Insomnia**	**Depression**	**Anxiety**
**Characteristic**	***N*** = **10,413**	***N*** = **1,900**	***N*** = **2,364**	***N*** = **1,335**
**Age group**				
18–29	3,570 (34.28%)	639 (33.63%)	814 (34.43%)	449 (33.63%)
30–44	1,548 (14.87%)	994 (52.32%)	293 (12.39%)	153 (11.46%)
45–60	5,295 (50.85%)	267 (14.05%)	1,257 (53.17%)	733 (54.91%)
**Gender**				
Male	7,718 (74.12%)	495 (26.05%)	1,795 (75.93%)	1,029 (77.08%)
Female	2,695 (25.88%)	1,405 (73.95%)	569 (24.07%)	306 (22.92%)
**Marital status**				
Married	6,594 (63.32%)	1,165 (61.32%)	1,440 (60.91%)	831 (62.25%)
Unmarried/divorced/widowed	3,819 (36.68%)	735 (38.68%)	924 (39.09%)	504 (37.75%)
**Education attainment**				
High school or below	205 (1.97%)	31 (1.63%)	28 (1.18%)	21 (1.57%)
University	1,744 (16.75%)	303 (15.95%)	365 (15.44%)	208 (15.58%)
Postgraduate or above	8,464 (81.28%)	1,566 (82.42%)	1,971 (83.38%)	1,106 (82.85%)
**Healthy lifestyle score**				
0	179 (1.72%)	45 (2.37%)	50 (2.12%)	23 (1.72%)
1	896 (8.60%)	199 (10.47%)	275 (11.63%)	152 (11.39%)
2	3,696 (35.49%)	689 (36.26%)	864 (36.55%)	486 (36.40%)
3	5,476 (52.59%)	951 (50.05%)	1,150 (48.65%)	661 (49.51%)
4	166 (1.59%)	16 (0.84%)	25 (1.06%)	13 (0.97%)
**Income satisfaction**				
High	2,741 (26.32%)	285 (15%)	340 (14.38%)	181 (13.56%)
Moderate	4,860 (46.67%)	843 (44.37%)	1,092 (46.19%)	596 (44.64%)
Low	2,812 (27.00%)	772 (40.63%)	932 (39.42%)	558 (41.80%)
**Monthly income**				
< 5,000	1,407 (13.51%)	225 (11.84%)	293 (12.39%)	182 (13.63%)
5,000–10,000	4,730 (45.42%)	884 (46.53%)	1,093 (46.24%)	631 (47.27%)
10,001–20,000	3,829 (36.77%)	730 (38.42%)	906 (38.32%)	483 (36.18%)
>20,000	447 (4.29%)	61 (3.21%)	72 (3.05%)	39 (2.92%)
**Work load**				
Low	1,239 (11.90%)	102 (5.37%)	117 (4.95%)	68 (5.09%)
Moderate	5,569 (53.48%)	722 (38%)	962 (40.69%)	485 (36.33%)
High	3,605 (34.62%)	1,076 (56.63%)	1,285 (54.36%)	782 (58.58%)
**Job category**				
Clinicians	2,105 (20.22%)	593 (31.21%)	390 (16.50%)	223 (16.70%)
Nurses	3,217 (30.89%)	1,010 (53.16%)	727 (30.75%)	397 (29.74%)
Paramedical staff	5,091 (48.89%)	297 (15.63%)	1,247 (52.75%)	715 (53.56%)
**Mental health training**	2,626 (25.22%)	343 (18.05%)	436 (18.44%)	235 (17.60%)
**Negative life events**	1,150 (11.04%)	257 (13.53%)	351 (14.85%)	188 (14.08%)
**Interpersonal relationship**				
Poor	131 (1.26%)	75 (3.95%)	105 (4.44%)	81 (6.07%)
Moderate	2,477 (23.79%)	761 (40.05%)	1,037 (43.87%)	637 (47.72%)
Good	7,805 (74.95%)	1,064 (56%)	1,222 (51.69%)	617 (46.22%)
**Verbal violence**				
0 times	6,243 (59.95%)	787 (41.42%)	986 (41.71%)	516 (38.65%)
1–3 times	3,245 (31.16%)	759 (39.95%)	969 (40.99%)	547 (40.97%)
4–6 times	519 (4.98%)	166 (8.74%)	209 (8.84%)	139 (10.41%)
7–9 times	109 (1.05%)	43 (2.26%)	49 (2.07%)	30 (2.25%)
≥10 times	297 (2.85%)	145 (7.63%)	151 (6.39%)	103 (7.72%)
**Physical violence**				
0 times	9,742 (93.56%)	1,698 (89.37%)	2,113 (89.38%)	1,177 (88.16%)
1–3 times	566 (5.44%)	165 (8.68%)	205 (8.67%)	137 (10.26%)
4–6 times	65 (0.62%)	19 (1.00%)	27 (1.14%)	12 (0.90%)
7–9 times	16 (0.15%)	7 (0.37%)	7 (0.30%)	4 (0.30%)
≥10 times	24 (0.23%)	11 (0.58%)	12 (0.51%)	5 (0.37%)
**Violence type**				
None	6,139 (58.96%)	767 (40.37%)	957 (40.48%)	500 (37.45%)
Verbal violence	3,603 (34.60%)	931 (49.00%)	1,156 (48.90%)	677 (50.71%)
Physical violence	104 (1.00%)	20 (1.05%)	29 (1.23%)	16 (1.20%)
Verbal violence & physical violence	567 (5.45%)	182 (9.58%)	222 (9.39%)	142 (10.64%)

### Association between WPV from patients and insomnia, depression, and anxiety

**Figure 1:**
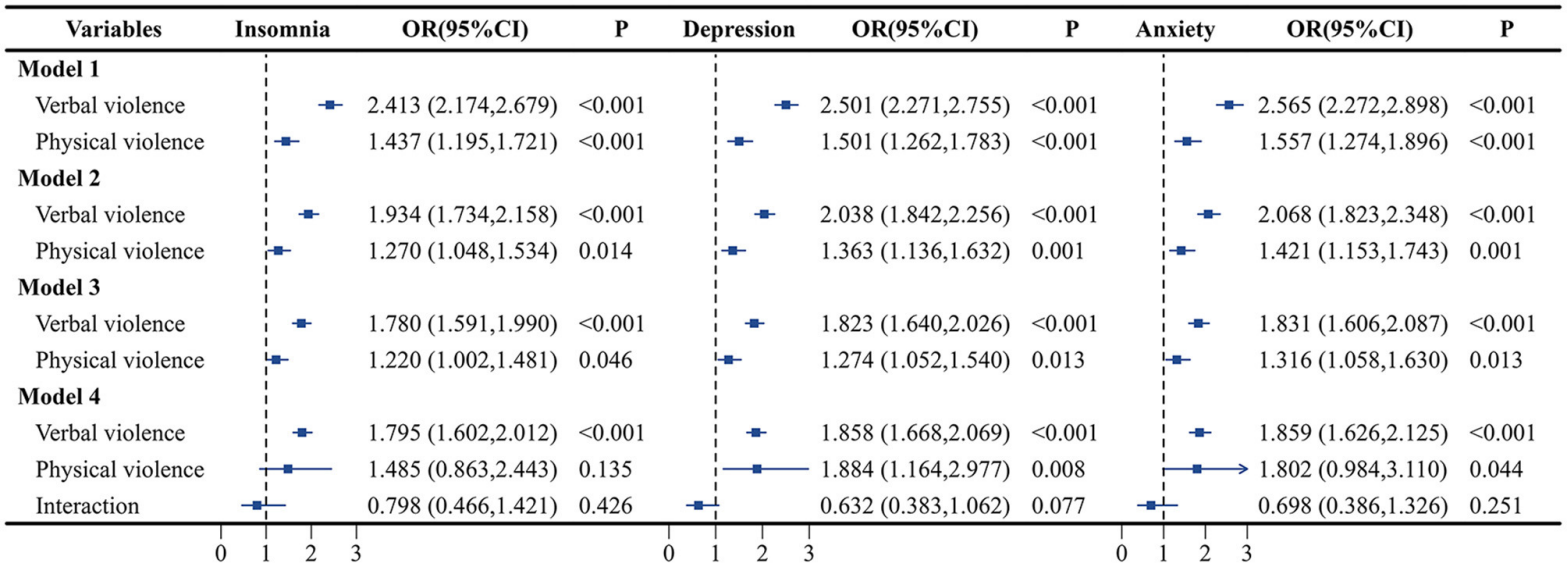
Association of verbal violence and physical violence with insomnia, depression, and anxiety. Model 1: adjust for sex, age group, marital status, education attainment. Model 2: adjust for the covaries in model 1 plus income satisfaction, monthly income, workload, and job category. Model 3: adjust for the covaries in model 2 plus healthy lifestyle score, mental health training, negative life events, and interpersonal relationships.

In the full-adjusted model, verbal violence and physical violence were associated with a higher risk of insomnia, depression, and anxiety. Compared with those who did not experience verbal violence, those who experienced verbal violence had a 78.0% increased risk of insomnia (OR = 1.780, 95% CI: 1.591–1.990), an 82.3% increased risk of depression (OR = 1.823, 95% CI: 1.640–2.026), and an 83.1% increased risk of anxiety (OR = 1.831, 95% CI: 1.606–2.087). Compared with those who did not experience physical violence, participants who experienced physical violence had a 22.0% increased risk of insomnia (OR = 1.220, 95% CI: 1.002–1.481), a 27.4% increased risk of depression (OR = 1.274, 95% CI: 1.052–1.540), and a 31.6% increased risk of anxiety (OR = 1.316, 95% CI: 1.058–1.630). Analysis of the interaction between physical violence and verbal violence revealed no significant interaction between the two forms of violence (*P* = 0.251), however, upon inclusion of the interaction term for verbal violence and physical violence, physical violence was only associated with a higher risk of depression and anxiety, with no significant association with insomnia observed. The results of four models between WPV among patients and the mental health status of medical staff are shown in Figure 1.

### Association between insomnia and depression and anxiety

Depression and anxiety were associated with an increased risk of insomnia. The likelihood of insomnia occurring with depressive symptoms is 7.606 times higher than those without depressive symptoms (OR = 7.606, 95% CI: 6.601–8.764). Similarly, compared with those who did not have anxiety symptoms, those who had anxiety symptoms had a 110.3% increased risk of insomnia (OR = 2.103, 95% CI: 1.792–2.467). Detailed information can be found in Table 2.

**Table 2:** Association of depression and anxiety with insomnia.

	**Insomnia**	
**Variables**	**OR (95% CI)**	***P***
**Depression**		
Model 1	4.626 (4.296–4.981)	< 0.001
Model 2	4.319 (4.004–4.658)	< 0.001
Model 3	4.281 (3.957–4.631)	< 0.001
**Anxiety**		
Model 1	4.286 (3.960–4.639)	< 0.001
Model 2	3.904 (3.601–4.233)	< 0.001
Model 3	3.736 (3.437–4.062)	< 0.001

Model 1: adjust for sex, age group, marital status, education attainment. Model 2: adjust for the covaries in model 1 plus income satisfaction, monthly income, workload, and job category. Model 3: adjust for the covaries in model 2 plus healthy lifestyle score, mental health training, negative life events, interpersonal relationships.

### The mediating effect of depression and anxiety between WPV from patients and insomnia

**Figure 2:**
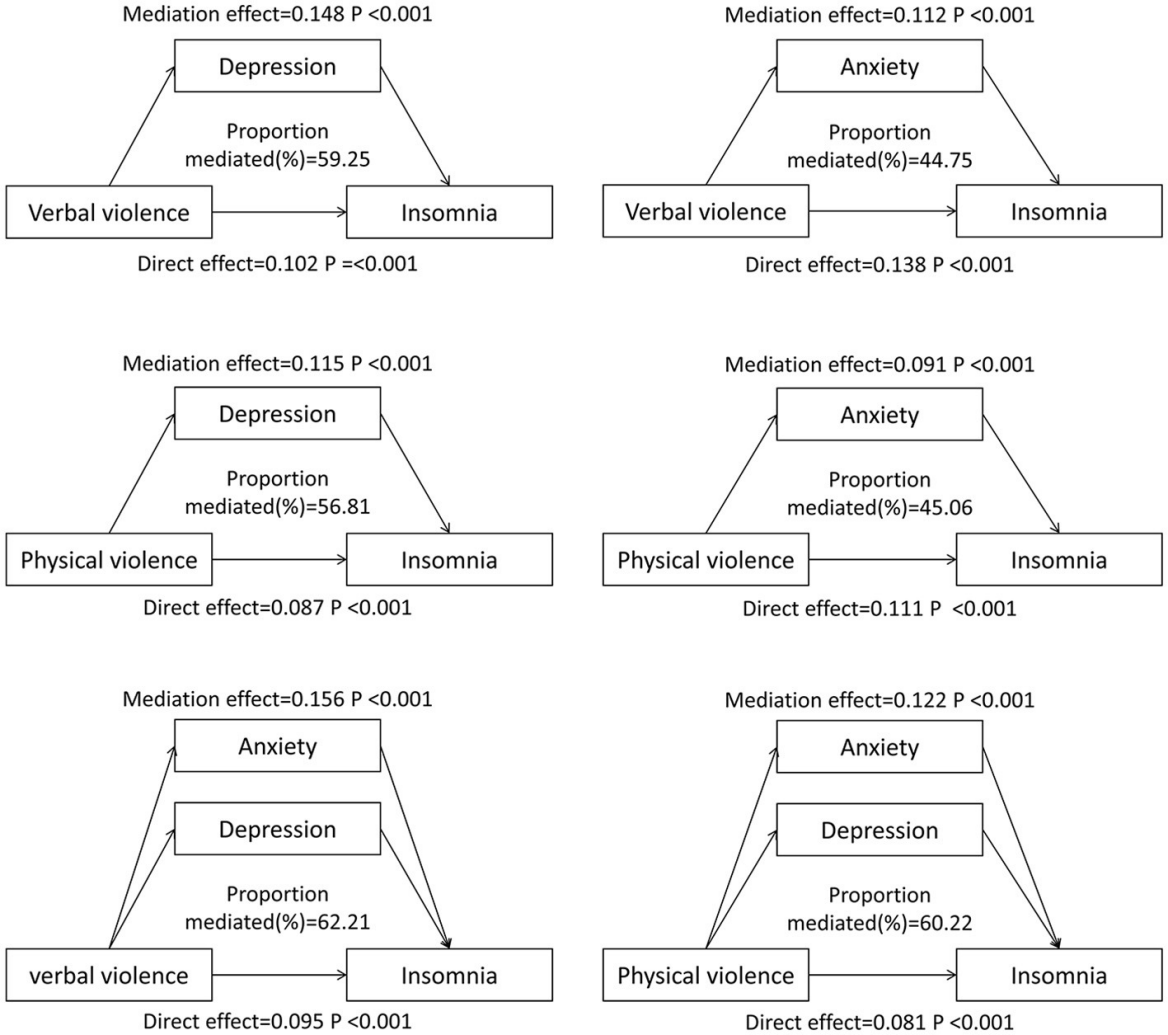
Mediating effects of healthy lifestyles in workplace violence and mental health. Models adjusted for sex, age group, marital status, education attainment, healthy lifestyle score, income satisfaction, monthly income, workload, job category, mental health training, negative life events, and interpersonal relationships.

Mediation analyses showed that depression and anxiety had significant mediating effects on the association between WPV from patients and insomnia of healthcare workers. The proportion of mediation of depression and anxiety between verbal violence and insomnia is 59.25% and 44.75%, respectively, while the proportion of mediation between physical violence and insomnia is 56.81% and 45.06%, respectively (both *P* < 0.001). Moreover, when depression and anxiety were both included in the mediation model, the mediated proportion was 62.21% in the association between verbal violence and insomnia, and 60.22% in the association between physical violence and insomnia. Detailed information can be found in Figure 2.

**Figure 3:**
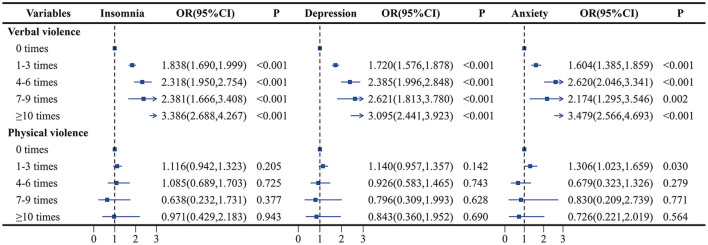
Association of different frequencies of verbal violence and physical violence with insomnia, depression, and anxiety. Adjust for sex, age group, marital status, education attainment, healthy lifestyle score, income satisfaction, monthly income, workload, job category, mental health training, negative life events, and interpersonal relationships.

We further evaluated the association between different frequencies of violence and the risk of insomnia, depression, and anxiety, a higher frequency of verbal violence was associated with a higher risk of insomnia, depression, and anxiety. Compared with those who did not experience verbal violence, those who experienced 1–3 times verbal violence had a 69.4 percent increased risk of insomnia (OR = 1.694, 95% CI: 1.556–1.844), a 108.7 percent increased risk of depression (OR = 2.087, 95% CI: 1.886–2.310), and a 72.0 percent increased risk of anxiety (OR = 1.720, 95% CI: 1.576–1.878). While after adjusting for verbal violence and other covariates, physical violence was not significantly associated with insomnia, depression, or anxiety. The details of the association between WPV from patients and the mental health status of healthcare workers in the full-adjusted model are shown in Figure 3.

Furthermore, the association between different types of violence and the risk of insomnia, depression, and anxiety was evaluated (Supplementary Table S1). Compared to participants who have not experienced any violence, experiencing any type of violence is associated with a higher risk of insomnia, depression, and anxiety. After adjustment for confounders, compared to those who have not experienced violence, those who have only experienced verbal violence have a 1.766 times higher risk of insomnia (OR = 1.766, 95% CI: 1.576–1.980), 1.858 times higher risk of depression (OR = 1.858, 95% CI: 1.668–2.069), 1.859 times higher risk of anxiety (OR = 1.859, 95% CI: 1.626–2.125), those who have only experienced physical violence have 1.465 times higher risk of insomnia (OR = 1.465, 95% CI: 0.852–2.411), 1.884 times higher risk of depression (OR = 1.884, 95% CI: 1.164–2.977), and 1.802 times higher risk of anxiety (OR = 1.802, 95% CI: 0.984–3.110). Those who have experienced both have a 2.123 times higher risk of insomnia (OR = 2.123, 95% CI: 1.716–2.620), 2.211 times higher risk of depression (OR = 2.211, 95% CI: 1.798–2.714), and 2.338 times higher risk of anxiety (OR = 2.338, 95% CI: 1.843–2.954).

Subgroup analyses of sex showed that verbal violence was associated with a higher risk of insomnia, depression, and anxiety in both males and females, and physical violence was significantly associated with an increased risk of depression in women (OR = 1.761, 95% CI: 1.029–2.933). The subgroup analysis results of job categories showed that verbal violence was associated with an increased risk of insomnia, depression, and anxiety among nurses, clinical doctors, and medical staff. However, physical violence was only associated with an increase in depression and anxiety risk among paramedical staff (OR = 3.307, 95% CI: 1.014–9.689, OR = 5.027, 95% CI: 1.408–15.493) (Supplementary Table S2).

## Discussion

A total of 10,413 healthcare workers were included in the study, with 18.25% reported insomnia, 40.05% reporting experiencing verbal violence and 6.44% reporting physical violence from patients in the past year. A total of 58.96% of participants did not experience any violence, while 5.45% experienced both types of violence. The study found that both verbal violence and physical violence were associated with an increased risk of insomnia, depression, and anxiety, with verbal violence showing greater significance. Moreover, a higher frequency of verbal violence was associated with elevated risks. The study also indicated that depression and anxiety played a significant mediating role in the association between WVP and insomnia.

The prevalence of WPV from patients in Zhuhai was lower at 41.04% compared to other regions in China such as Jiangsu Province (62.26%), Xinjiang Province (68.69%), Heilongjiang Province (64.88%), and the national average reported in a 2018 survey (65.3%) (35–38). This disparity in findings may be attributed to the composition of the survey subjects in this study, which included clinical doctors, nurses, and paramedical staff. Previous studies primarily focused on doctors and nurses in emergency departments, pediatrics, and other clinical departments, where the likelihood of experiencing WPV from patients is generally higher (39–41). A cross-sectional survey in China found that 82.4% of psychiatric nurses have experienced at least one violent event in the past 6 months, with a verbal violence incidence rate of 78.6% (42), which was higher than other studies. Furthermore, the economic prosperity and robust healthcare infrastructure in Zhuhai may play a role in mitigating violence rates within healthcare settings (43, 44). The incidence of WPV from patients in Zhuhai is comparable to the 44.6% WPV reported in Hong Kong in 2017, and the geographical location and economic level of the two places are relatively similar, with both regions sharing similar geographical locations and economic profiles (45). These findings emphasize the need for targeted interventions and further research to understand better the contextual, regional, and socio-economic influences on violence in healthcare environments.

Our study found that there is a significant association between physical and verbal violence and insomnia, depression, and anxiety among healthcare workers. This is in line with other studies demonstrating the detrimental impact of WPV on mental wellbeing (46–49). A systematic review focusing on WPV against nurses highlighted its potential to trigger various work-related and mental health consequences, including depression and anxiety (14). Similarly, a cross-sectional study in Taiwan demonstrated a significant association between psychiatric nurses who experienced patient attacks and depressive symptoms (50). These studies emphasized the need for effective interventions and support systems to mitigate the impact of WPV on mental health.

Interestingly, our study revealed that the association between verbal violence and mental health issues is more significant than that of physical violence, with a higher frequency of verbal violence increasing the risk of these mental health problems, and the interaction has no statistical significance. This suggests that verbal violence may have a deeper and longer-lasting impact on individuals, potentially leading to psychological trauma and mental health issues (51, 52). Zhan et al. found that physical violence was not significantly correlated with the adverse occupational outcomes of healthcare workers (53). This is similar to our results. As the most common form of WPV experienced by healthcare workers, frequent verbal violence can severely affect an individual's mental health, with its destructive effects potentially lasting for months or even years (46, 54, 55). However, there is a concerning lack of reporting of verbal violence incidents within the healthcare system, with a study showing that while 64.7% of nurses have encountered verbal violence, only 36.9% have reported it to their superiors, as this type of violence does not result in physical harm and they do not know how to report it or believe that hospitals tend to support patients more (56). Another possible reason might be due to the extremely low prevalence of physical violence reported by participants, which resulted in a less significant association between each frequency of physical violence and mental health issues. Still, the noteworthy adverse impact of non-physical patient violence on occupational outcomes warrants heightened scrutiny and robust intervention efforts from policymakers and law enforcement authorities at the national, provincial, and local levels, as well as from hospital administrators. Moreover, our research underscores the importance of future investigations delving into various manifestations of violence perpetrated by patients and their families or friends.

The mediation analysis revealed that depression and anxiety played a mediating role in the association between verbal violence and insomnia. Verbal violence was linked to heightened risks of depression and anxiety, subsequently increasing the likelihood of insomnia. Stressful events can trigger physiological and psychological stress responses in individuals (57–59). Incidents of WPV that jeopardize safety may activate individual defense mechanisms, resulting in tension, anxiety, and coping difficulties (6, 60). The accumulation of psychological pressure and negative emotions directly manifests as depression and anxiety, impacting an individual's quality of sleep. Many studies have proven insomnia is an important symptom of depression and anxiety (18, 19, 61, 62). Additionally, insomnia can prevent the body from receiving sufficient rest and recovery, thereby affecting an individual's emotional and psychological state, exacerbating symptoms of depression and anxiety, and forming a vicious cycle (58, 63). The existence of this mediating effect further emphasizes the importance of depression and anxiety in the impact of WPV on insomnia and provides a new perspective for further exploring the relationship between WPV, depression, anxiety, and insomnia.

This study has several strengths. First, this study has a large sample size and rich variables. Second, this study discussed in detail the impact of different frequencies of violence on the mental health of healthcare workers and discussed the mediating role of healthy lifestyles for the first time in China. However, there are also some limitations. Firstly, the study only surveyed healthcare workers in all secondary and tertiary hospitals in Zhuhai, which limits the generalizability of the results. Secondly, the study collected basic information and past year violence experiences of healthcare workers through self-reporting, which may lead to recall bias. Thirdly, this study did not investigate the experience of healthcare workers being subjected to WPV from patients' family members, which may have led to an underestimate of the incidence of violent incidents, and the severity of the consequences. Fourthly, as this study is a cross-sectional design, it cannot determine the causal relationship between WPV from patients and psychological health.

## Conclusion

In conclusion, this study reveals a significant association between WPV from patients and the mental health issues (insomnia, depression, and anxiety) of healthcare workers. This finding underscores the need for public health authorities to address the problem of WPV from patients, as it may adversely affect the mental wellbeing of healthcare workers and have implications for the quality of medical services and patient safety (64). Healthcare administrators should implement a series of preventive measures, including staff training, patient education, reporting mechanisms, and psychological support, to improve healthcare workers' mental health through coordinated efforts (65–67). Furthermore, the mediation analysis underscores the role of depression and anxiety in the relationship between verbal violence and insomnia. The existence of this mediating effect further emphasizes the importance of depression and anxiety in the impact of WPV on insomnia and provides a valuable perspective for further exploring the relationships among WPV, depression, anxiety, and insomnia. Future research should focus on the long-term effects of WPV on mental health, as well as the effectiveness of specific intervention measures, in order to deepen our understanding of WPV and its impact on healthcare workers.

## Data Availability

The data analyzed in this study is subject to the following licenses/restrictions: The data that support the findings of this study are available from the corresponding author upon reasonable request. Requests to access these datasets should be directed to Yongyong Teng, gztengyy@163.com.
