# Supplementary material for: Association between workplace violence from patients and the mental health status of healthcare workers in Zhuhai China: a cross-sectional study
Source: Front Public Health. 2025 Jan 7;12:1441389. doi: 10.3389/fpubh.2024.1441389 (PMC11747547; doi:10.3389/fpubh.2024.1441389)
Supplement: Supplementary file 1 [file Data_Sheet_1.docx]

Supplementary Material

Association between workplace violence from patients and the mental health status of healthcare workers in Zhuhai China: A cross-sectional study

**Ying Zheng1 2&, Xuping Li 1 2&, Yajun Sun2, Jingya Li1, Xiaohui Wang2, Yongyong Teng2***

*** Correspondence:** Yongyong Teng, gztengyy@163.com;

# Supplementary Figures and Tables

## Supplementary Tables

**Supplementary Table1.** The association of different types of workplace violence with mental health.

| Outcomes | None violence | Verbal violence | | Physical violence | | Verbal violence & Physical violence | |
| --- | --- | --- | --- | --- | --- | --- | --- |
|  | OR (95% CI) | OR (95% CI) | *P* | OR (95% CI) | *P* | OR (95% CI) | *P* |
| Insomnia | 1 (Ref.) | 1.766(1.576-1.980) | <0.001 | 1.465(0.852-2.411) | 0.149 | 2.123(1.716-2.620) | <0.001 |
| Depression | 1 (Ref.) | 1.858(1.668-2.069) | <0.001 | 1.884(1.164-2.977) | 0.008 | 2.211(1.798-2.714) | <0.001 |
| Anxiety | 1 (Ref.) | 1.859(1.626-2.125) | <0.001 | 1.802(0.984-3.110) | 0.044 | 2.338(1.843-2.954) | <0.001 |

*Adjust for sex, age group, marital status, education attainment, healthy lifestyle score, income satisfaction, monthly income, workload, job category, mental health training, negative life events, interpersonal relationship.

**Supplementary Table2**. Association of workplace violence with mental health in subgroups.

|  |  | Verbal violence | | Physical violence | | Interaction | |  |
| --- | --- | --- | --- | --- | --- | --- | --- | --- |
|  |  | OR (95% CI) | *P* | OR (95% CI) | *P* | OR (95% CI) | *P* | |
| **Insomnia** | **ALL participants** | 1.795(1.602,2.012) | <0.001 | 1.485(0.863,2.443) | 0.135 | 0.798(0.466,1.421) | 0.426 | |
|  | **Sex** |  |  |  |  |  |  | |
|  | Male | 2.233(1.774,2.814) | <0.001 | 1.493(0.338,4.656) | 0.536 | 0.712(0.213,3.278) | 0.615 | |
|  | Female | 1.664(1.459,1.899) | <0.001 | 1.452(0.798,2.515) | 0.185 | 0.847(0.465,1.609) | 0.573 | |
|  | **Job category** |  |  |  |  |  |  | |
|  | Clinicians | 1.854(1.515,2.273) | <0.001 | 1.193(0.180,4.636) | 0.2 | 1.190(0.285,8.188) | 0.598 | |
|  | Nurses | 1.775(1.513,2.082) | <0.001 | 1.319(0.683,2.393) | 0.383 | 0.837(0.438,1.686) | 0.602 | |
|  | Paramedical Staff | 1.689(1.277,2.251) | <0.001 | 2.342(0.602,7.341) | 0.173 | 0.573(0.144,2.638) | 0.446 | |
|  |  |  |  |  |  |  |  | |
| **Depression** | **ALL participants** | 1.858(1.668,2.069) | <0.001 | 1.884(1.164,2.977) | 0.008 | 0.632(0.383,1.062) | 0.077 | |
|  | **Sex** |  |  |  |  |  |  | |
|  | Male | 1.816(1.451,2.275) | <0.001 | 2.791(0.866,7.572) | 0.059 | 0.591(0.203,2.006) | 0.361 | |
|  | Female | 1.862(1.646,2.106) | <0.001 | 1.761(1.029,2.933) | 0.033 | 0.615(0.351,1.102) | 0.095 | |
|  | **Job category** |  |  |  |  |  |  | |
|  | Clinicians | 1.831(1.511,2.222) | <0.001 | 2.263(0.583,7.305) | 0.195 | 0.610(0.175,2.515) | 0.459 | |
|  | Nurses | 1.911(1.643,2.225) | <0.001 | 1.627(0.898,2.840) | 0.096 | 0.670(0.364,1.270) | 0.206 | |
|  | Paramedical Staff | 1.710(1.315,2.220) | <0.001 | 3.307(1.014,9.689) | 0.035 | 0.583(0.160,2.293) | 0.423 | |
|  |  |  |  |  |  |  |  | |
| **Anxiety** | **ALL participants** | 1.859(1.626,2.125) | <0.001 | 1.802(0.984,3.110) | 0.044 | 0.698(0.386,1.326) | 0.251 | |
|  | **Sex** |  |  |  |  |  |  | |
|  | Male | 1.779(1.337,2.373) | <0.001 | 2.020(0.310,7.503) | 0.363 | 0.964(0.242,6.503) | 0.964 | |
|  | Female | 1.896(1.631,2.206) | <0.001 | 1.790(0.964,3.325) | 0.065 | 0.634(0.324,1.239) | 0.183 | |
|  | **Job category** |  |  |  |  |  |  | |
|  | Clinicians | 1.884(1.472,2.419) | <0.001 | 3.710(0.786,13.084) | 0.059 | 0.438(0.114,2.197) | 0.262 | |
|  | Nurses | 1.899(1.579,2.286) | <0.001 | 1.128(0.482,2.324) | 0.761 | 1.065(0.491,2.591) | 0.881 | |
|  | Paramedical Staff | 1.769(1.278,2.443) | 0.001 | 5.027(1.408,15.493) | 0.007 | 0.231(0.056,1.028) | 0.046 | |

*Adjust for sex, age group, marital status, education attainment, healthy lifestyle score, income satisfaction, monthly income, workload, job category, mental health training, negative life events, interpersonal relationship.
